# Draft Genome Sequence of Lipopeptide-Producing Strain Pseudomonas fluorescens DSM 11579 and Comparative Genomics with *Pseudomonas* sp. Strain SH-C52, a Closely Related Lipopeptide-Producing Strain

**DOI:** 10.1128/MRA.00304-20

**Published:** 2020-05-21

**Authors:** Norbert Kirchner, Carolina Cano-Prieto, Menno van der Voort, Jos M. Raaijmakers, Harald Gross

**Affiliations:** a Department of Pharmaceutical Biology, Pharmaceutical Institute, University of Tübingen, Tübingen, Germany; b German Centre for Infection Research (DZIF), Partner Site Tübingen, Tübingen, Germany; c Department of Microbial Ecology, Netherlands Institute of Ecology (NIOO-KNAW), Wageningen, The Netherlands; d Institute of Biology, Leiden University, Leiden, The Netherlands; Loyola University Chicago

## Abstract

Pseudomonas fluorescens DSM 11579 is known to be a producer of the lipopeptides brabantamide and thanamycin. Its draft genome gives insight into the complete secondary metabolite production capacity of the strain and builds the basis for a comparative study with *Pseudomonas* sp. strain SH-C52, a lipopeptide-producing strain involved in natural disease-suppressive soils.

## ANNOUNCEMENT

As part of our ongoing efforts to investigate plant growth-promoting rhizobacteria (PGPR), one of the authors (J.M.R.) isolated the plant-associated strain *Pseudomonas* sp. SH-C52 from a soil suppressive to damping-off disease caused by the fungal root pathogen Rhizoctonia solani (1). Recently, we reported that SH-C52 produces the bioactive lipopeptides thanapeptin and thanamycin and also the cyclocarbamate brabantamide A (syn. SB-253514), which all contribute to the disease-suppressive effect of this strain (2–4). The latter two compound groups are also produced by the strain Pseudomonas fluorescens DSM 11579 (5–7), but the corresponding genomic data were not publicly available. In order to enable comparative genomic and taxonomic analyses, the sequencing of strain DSM 11579 was initiated.

The strain DSM 11579 was obtained from the German Collection of Microorganisms and Cell Cultures (DSMZ). The strain was subcultured to purity and analyzed using 16S rRNA, and pure cultures were stored at −80°C in 2 × 1.5 ml Trypticase soy broth (TSB) with 50% glycerol, employing 2-ml cryogenic vials (Nalgene). For genomic DNA (gDNA) extraction, 150 μl of the preserved pure strain was used to inoculate 15 ml of TSB and was grown at 20°C on a rotary shaker (120 rpm). After 48 h, cells were harvested, and the gDNA was isolated as previously described (8). The genome of DSM 11579 was sequenced using a combined Illumina/PacBio sequencing approach. An aliquot of the obtained gDNA was used for Illumina HiSeq 2500 sequencing. Upon Nextera-XT paired-end library preparation, gDNA was first subjected to 2 × 125-cycle paired-end sequencing, producing 3,362,456 reads. FASTQ sequence files were generated using the Illumina CASAVA pipeline v1.8.3. Initial quality assessment was based on data passing the Illumina chastity filtering. Subsequently, reads containing PhiX control signal were removed. In addition, reads containing (partial) adapters were clipped (up to a minimum read length of 50 bp). The second quality assessment was based on the remaining reads using the FastQC quality control tool v0.10.0 (9). The quality of the Illumina FASTQ sequences was enhanced by trimming off low-quality bases using the program bbduk, which is part of the BBMap suite v34.46 (10). Subsequently, the filtered reads were assembled into contig sequences using ABySS v1.5.1.

The remaining gDNA was sheared to 10 kb and used to generate SMRTbell libraries using the standard library protocols of the Pacific Biosciences DNA template preparation kit. The finished library was bound to P4 polymerase and sequenced on a PacBio RS sequencer using C2 chemistry (1 single-molecule real-time [SMRT] cell). The data collected were processed and filtered using the SMRT Analysis software suite. The continuous long-read (CLR) data were filtered by read length (>35 bp), subread length (>35 bp), and read quality (>0.75). The final quality statistics included 344,142 reads with an average read length of 3,534 bp and a maximum read length of 38,722 bp. The Illumina-based contigs were aligned against the PacBio CLR reads using BLASR v1 (11). Based on the alignment, contigs were placed into superscaffolds using the SSPACE-LongRead scaffolder v1.0 (12). The gapped regions within the superscaffolds were closed using Gap-Filler v1.10 (13). Software parameter settings were kept at the defaults, unless stated otherwise. The final assembly yielded one scaffold made of two contigs for a total of 6,132,423 bp. The assembly has an average coverage of 129-fold and exhibits a G+C content of 61.8%. Gene predictions and annotations were provided by NCBI using the NCBI Prokaryotic Genome Annotation Pipeline (PGAP) v4.11 (14), yielding a total of 5,249 coding genes.

An automated genome-based taxonomic analysis of DSM 11579, employing TYGS (15), revealed that the initial taxonomic classification of strain DSM 11579 was not correct. The cormycin/corpeptin-producing strain Pseudomonas mediterranea CFBP5447^T^ (16) was identified as its closest related type strain. In pairwise comparisons, the digital DNA-DNA hybridization (DDH) values (d_4_) between strain DSM 11579 and its closest related type strains ranged from 23.1 to 36.0%. Since these values are below the species threshold of 70%, DSM 11579 represents a new *Pseudomonas* species. Further bioinformatic analyses using antiSMASH v5.1 (17) revealed that strain DSM 11579 possesses to a large extent the same type of biosynthetic gene clusters (BGCs) as SH-C52. These include a pyoverdine, a set of octa-/nona-/docosa-lipopeptides (2), brabantamide (3), bacteriocins, a beta-lactone, NAGGN (18), an aryl polyene (19), and a fragin-like compound (20). However, the biosynthesis of these pathways may be regulated differently in DSM 11579, since it possesses, in addition to the above-mentioned BGCs, a homoserine (21) and a phenazine (22) BGC, while SH-C52 contains an additional BGC encoding the siderophore achromobactin (23), which may enable it to compete better under iron-limited conditions.

### Data availability.

This whole-genome sequencing (WGS) project has been deposited at DDBJ/ENA/GenBank under the accession number JAAOIQ000000000. The raw sequencing data sets have been registered in the NCBI SRA database under the accession numbers SRR11306406 and SRR11306407.
